# Effect of *Trichoderma*-enriched organic charcoal in the integrated wood protection strategy

**DOI:** 10.1371/journal.pone.0183004

**Published:** 2017-08-10

**Authors:** Javier Ribera, Mónica Gandía, Jose F. Marcos, Maria del Carmen Bas, Siegfried Fink, Francis W. M. R. Schwarze

**Affiliations:** 1 Department of Applied Wood Materials, Empa, St. Gallen, Switzerland; 2 Professur für Forstbotanik, Albert-Ludwigs-Universität Freiburg, Freiburg im Breisgau, Germany; 3 Department of Food Biotechnology, Instituto de Agroquímica y Tecnología de Alimentos, Consejo Superior de Investigaciones Científicas, Paterna, Spain; 4 Department of Applied Statistics and Operational Research and Quality, Universitat Politècnica de València, València, Spain; USDA Forest Service, UNITED STATES

## Abstract

The gradual elimination of chromium from wood preservative formulations results in higher Cu leaching and increased susceptibility to wood decay fungi. Finding a sustainable strategy in wood protection has become of great interest among researchers. The objective of these *in vitro* studies was to demonstrate the effect of T-720-enriched organic charcoal (biochar) against five wood decay basidiomycetes isolated from strongly damaged poles. For this purpose, the antagonistic potential of *Trichoderma harzianum* (strain T-720) was confirmed among other four *Trichoderma* spp. against five brown-rot basidiomycetes in dual culture tests. T-720 was genetically transformed and tagged with the green fluorescent protein (GFP) in order to study its antagonistic mechanism against wood decay basidiomycetes. It was also demonstrated that T-720 inhibits the oxalic acid production by basidiomycetes, a well-known mechanism used by brown-rot fungi to detoxify Cu from impregnated wood. Additionally, this study evaluated the effect of biochar, alone or in combination with T-720, on Cu leaching by different preservatives, pH stabilization and prevention of wood decay caused by five basidiomycetes. Addition of biochar resulted in a significant Cu binding released from impregnated wood specimens. T-720-enriched biochar showed a significant reduction of wood decay caused by four basidiomycetes. The addition of T-720-enriched biochar to the soil into which utility poles are placed may improve the efficiency of Cr-free wood preservatives.

## Introduction

Wood is still one of the most used construction material due to its abundancy, production costs and environmental benefits. However, as wood is biodegradable it has a limited service life and is a mandatory requirement to impregnate wood products in ground contact with copper (Cu)-based wood preservatives that are effective against a range of soil microorganisms [1]. The increasing interest to protect the environment resulted in the phase out of the traditional wood preservative formulations that contained strongly carcinogenic compounds such as arsenic, fluor or chromium (Cr) [2]. The absence of Cr results in higher Cu leaching from impregnated wood even after short periods of installation in the ground [2–5]. The released Cu, as inorganic compound, is not subjected to biological degradation and it is therefore persisting in the environment affecting bioaccumulation and producing toxicity [6]. Moreover, the continuous use of Cu-based wood preservatives has resulted in the development of resistance in a range of wood decay fungi [7], through the production of oxalic acid [8–13]. The resulting Cu oxalate complex loses its toxicity properties as a fungicide which results in a reduction in the service life of the wood products [14, 15].

Alternative management strategies to improve and prolong the service life of wood products have resulted in great interest due to healthy and environmental reasons [16–18]. The possibility to develop an integrated wood protection method has been evaluated by several authors that studied the effect of biological control agents against wood decay fungi [19–21]. In recent laboratory studies, the possibility to use an integrated control strategy combining a biocontrol agent (*Trichoderma* spp.) with low concentrations of Cr-free wood preservatives was demonstrated [22]. Thus, *Trichoderma harzianum* (strain T-720) showed a strong tolerance to Cu-amended medium (up to 0.1% of CuSO_4_) and a high antagonistic potential in combination with a range of wood preservative formulations against three wood decay basidiomycetes [15, 22].

In the last decades, charcoal (biochar) has been used as soil amendment to improve soil properties and increase agriculture productivity. The application of biochar in soils can be beneficial as it results in increase surface areas, retention of water and heavy metals, stabilisation of pH and carbon sequestration in different substrates [23, 24]. Moreover, many biochar-associated components have biocidal activity which increases the stability against soil microorganisms. During the process of biochar production from organic matter, the pyrolization shifts the chemical composition of the raw material into condensed aromatic structures that improve long term properties [25]. In addition, biochar has been postulated as potential bioremediation method for polluted soils [23]. These biochar properties may play an important role in improving the efficiency of Cr-free wood preservatives.

The main aim of this study was to confirm the antagonistic potential of T-720 and evaluate its potential to control the oxalic acid production by wood decay basidiomycetes. In addition, we examined the capacity of biochar to bind Cu released from impregnated wood specimens exposed to leaching. And finally, the integrated control potential of T-720-enriched biochar against wood decay basidiomycetes was also evaluated.

## Materials and methods

### Antagonistic potential of *Trichoderma* against wood decay basidiomycetes

The antagonistic potential of strain T-720, used in previous studies, was confirmed in dual cultures against five wood decay basidiomycetes. For this purpose, agar discs (5 mm) of cultures of wood decay basidiomycetes (Table 1) were inoculated on one side of the Petri dish containing 2% malt extract agar (MEA) (Oxoid, Pratteln, Switzerland) and incubated at 22(±1)°C and 70% relative humidity. After 10 days, the opposite side of the Petri dish was inoculated with 100 μL of *Trichoderma* spp. (Table 1) spores suspension adjusted to 10^6^ spores mL^-1^ [22]. Five Petri dishes (biological replicates) for each combination of *Trichoderma* strains and basidiomycetes were evaluated. Four weeks after the inoculation of *Trichoderma*, the overgrowth, sporulation tufts and pustules of *Trichoderma* strains on the basidiomycetes were used to evaluate its activity [26]. The rate of mycoparasitism in dual cultures was assessed as + = slow overgrowth, ++ = fast overgrowth, +++ = very fast overgrowth. In order to check whether the *Trichoderma* species and strains were able to parasitize and eradicate the challenged basidiomycete, five agar discs (5 mm) were removed from non-sporulating regions of the basidiomycete and placed on a basidiomycete’s selective medium containing 20 mL of 2% MEA with 2 mL of thiabendazole dissolved in lactic acid (Merck, Darmstadt, Germany) [27]. If the basidiomycete failed to grow on thiabendazole amended medium, the lethal effect of *Trichoderma* was considered to be 100% [22, 28].

**Table 1:** Fungi used in the present study.

Basidiomycete	Strain	*Trichoderma* species and strains	Strain
***Antrodia serialis***	A-481	*T*. *atroviride* Karsten	T-685
***Antrodia vaillantii***	F-774	*T*. *harzianum*	T-720
***Gloeophyllum sepiarium***	G-592	*T*. *harzianum*	T-721
***Rhodonia placenta***	Empa 45^1^	*T*. *atroviride*	T-722
***Serpula himantioides***	S-731	*T*. *koningiopsis*	T-723

^1^EMPA collection; Swiss Federal Laboratories for Material Science and Technology. St. Gallen, Switzerland.

### *Trichoderma harzianum* (T-720) and oxalic acid production of wood decay basidiomycetes

Dual cultures with wood decay basidiomycetes and T-720 (Table 1) were prepared in liquid culture with 120 mL of 1% malt (OXOID). One agar disc of fresh cultures of basidiomycetes were inoculated in 250 mL Erlenmeyer flasks and incubated in the dark at 25(±1)°C and 120 rpm on a shaker. After 20 days, 100 μL of spore suspension from fresh cultures of T-720 (10^6^ spores mL^-1^) were inoculated in the liquid medium containing the wood decay basidiomycetes. Three Erlenmeyer (biological replicates) for each combination of T-720 and basidiomycetes were evaluated. Three flasks for each wood decay basidiomycete were kept as controls. Four weeks after the *Trichoderma* treatment, the supernatant was filtered with 5 μm Millipore filters (Sigma-Aldrich, Buchs, Switzerland) and three aliquots (3 repetitions) of 500 μL were removed. Furthermore, the aliquots were centrifuged at 725 x *g* for 1 min to remove cell debris and the obtained solution was analyzed according to the Oxalic acid colorimetric assay kit (Sigma-Aldrich).

### Biochar and Cu-leaching from Cu-treated wood specimens

Wood specimens of Scots pine (*Pinus sylvestris* L.) sapwood (2 x 2 x 8 cm; radial, tangential, longitudinal) were separately impregnated with CC (copper-chromium), CCB (copper-chromium-boron), Cu-HDO (Bis-(N-cyclohexyldiazeniumdioxy)-copper) and ACQ (Alkaline Copper Quaternary) (Table 2) according to EN 252 [29]. The wood specimens were impregnated with concentrations of wood preservative that demonstrated toxic effect in previous studies by Ribera et al. [22] (Table 2). Afterwards, the wood specimens were placed into Erlenmeyer flasks with 500 mL of deionized water containing 2.5 g of sterilized biochar powder (Carbon Gold, Bristol, UK). The source of biochar was a commercial blend of hardwood species made at a pyrolysis temperature between 500–700°C. Three flasks for each combination of wood preservative and biochar treatment were evaluated. Duplicates without biochar were used as Cu-leaching controls. After 10 days immersed in water and water containing biochar, wood specimens were removed and the solutions were centrifuged at 700 x *g* for 30 min. The obtained supernatant was filtered with Whatman No. 1 filter paper (Sigma-Aldrich) and the solution was separated from the biochar. The pH in the supernatant was measured and then mixed with 5 mL of 2% HNO_3_ for directly quantification of Cu in solution using inductively coupled plasma optical emission spectrometry (ICP-OES). Additionally, the retention capacity of Cu by the removed biochar was analyzed. For this purpose, 1 g of the extracted biochar was mixed with 3 mL of 2% HNO_3_ and 1 mL of 35% H_2_O_2_. After 10 min digestion in the microwave at 500 Watt, the Cu content in solution was measured using ICP-OES.

**Table 2:** Wood preservative formulations and retentions of the impregnated wood specimens.

Formulation	Retention (kg m^-3^)
**Copper-Chromium (CC)**	21.3
**Copper-Chromium-Boron (CCB)**	40.3
**Chromium-free (Cu-HDO)**	43.3
**Chromium-free (ACQ)**	26.1

### *Trichoderma harzianum* (T-720)-enriched biochar and wood mass loss reduction by wood decay basidiomycetes

Interaction tests with wood block specimens of Scots pine sapwood (2.5R x 1.5T x 5L cm) were performed as described by Ribera et al. [22] with the following modifications. For evaluation of the effect of T-720 and biochar on reducing decay against wood decay basidiomycetes (Table 1) autoclavable plastic containers (WEZ, Oberentfelden, Switzerland. dimensions; 25L x 25W x 20H cm) with 180 g of vermiculite (VTT AG, Muttenz, Switzerland) and 5 g of Scots pine sawdust were used. The moisture content and water holding capacity of the substrate was determined according to ENV 807 [30]. The amount of water needed to bring the substrate to 75% of its water holding capacity was calculated and added to the containers. After autoclave sterilisation, three containers for each decay basidiomycete were inoculated (replicates) and used as controls. After 8 weeks incubation with the basidiomycetes, three wood specimens were sterilised with ethylene oxide and placed into each container. Determination of the initial wood dry mass was calculated by oven drying (103°C) test specimens during 24 h. Twelve weeks after incubation, the specimens were removed, oven dried (103°C) and the mass loss recorded. The influence of biochar on the basidiomycetes was evaluated in containers with 180 g of vermiculite, 50 g of biochar and 5 g sawdust. Three containers for basidiomycete were inoculated. And 8 weeks after inoculation, three wood specimens (repetitions) were placed into each container as described above. Twelve weeks later the specimens were removed and the mass loss was recorded. In order to study the effect of *Trichoderma*-enriched biochar on preventing wood decay, 50 g of biochar per container were incubated with 5 mL of T-720 spore suspension (10^6^ spores mL^-1^). After two weeks colonisation, the T-720-enriched biochar was added into the vermiculite boxes containing basidiomycetes as described above. After 2 weeks of the T-720-enriched biochar treatment, three specimens were placed into each container. Three boxes for each basidiomycete were treated with T-720-enriched biochar and 12 weeks later, the specimens were also removed and mass losses recorded.

### Generation of *T*. *harzianum* transformants

The pCAMBgfp binary vector containing the hygromycin B resistance gene and the *gfp* (green fluorescent protein) gene [31] was introduced into *Agrobacterium tumefaciens* AGL-1 for fungal *Agrobacterium tumefaciens*-mediated transformation (ATMT). The transformation of T-720 was conducted by ATMT essentially as described by Khang et al. [32] and Harries et al. [33]. After 48 h of co-culture of T-720 and AGL-1 (pCAMBgfp), the selection of transformants was conducted on PDA plates (potato dextrose agar, Difco BD Diagnostics, Sparks, MD, USA) amended with 50 μg mL^-1^ hygromicin (Invivogen, ant-hm-5 San Diego, CA, USA) incubated for 2 days at 24°C. The transformed colonies of T-720 expressing the *gfp* gene (T720G) were isolated and stored in aliquots of 2 mL containing 80% glycerol (Sigma-Aldrich) at -80°C for further studies. The transformants were confirmed by PCR (polymerase chain reaction) amplification of genomic DNA with oligonucleotide primers specific for the *gfp* gene (forward OJM469: 5'-CCACATGAAGCAGCACGACT-3', reverse OJM470: 5'-CTTCAGCTCGATGCGGTTC-3') and the hygromycin B resistance gene (forward OJM197: 5'-CGTTAACTGATATTGAAGGAGCAT-3', reverse OJM198: 5'-TGTTAACTGGTTCCCGGTCGG-3') according to Gandía et al. [34]. Additionally, the antagonistic potential of T-720G was evaluated in dual cultures against *Rhodonia placenta*, *Antrodia serialis* and *Serpula himantioides* to demonstrate the influence of the inserted *gfp* gene.

### Microscopy

The overgrowth of T-720G on the mycelium of the basidiomycetes was observed by confocal laser scanning microscopy (CLSM) (Zeiss LSM T-PMT). Fungal mycelium was collected from the contact area of dual cultures after 48 h of the first mutual contact. The samples were stained with 10 μL propidium iodide (Sigma-Aldrich) for 10 min to label the mycelium of the basidiomycetes. Microscopic preparations were visualized at excitation/emission of 488/550 mm wavelengths for the GFP and 600/750 mm for propidium iodide as described by Chacón et al. [35]. Additionally, interactions between the T-720G and biochar were analyzed by SEM (Hitachi S-4800) and fluorescence microscopy (Leica DM 4000 B LED), respectively. To study the colonisation of *Trichoderma* on the biochar substrate, 5 g of sterile biochar were inoculated with 5 mL of T-720G spore suspension (10^6^ spores mL^-1^). After 48 h of inoculation the colonised biochar was collected and directly observed with both microscopic techniques.

### Statistical analysis

To evaluate the effect of the different treatments compared to controls such as the influence of T-720 on the oxalic acid production, the Cu-retention and the influence on the pH by biochar, a t-test was applied. Besides, comparison between the basidiomycetes for oxalic acid production and the different wood preservative formulations for the Cu-retention assay were assessed by a Tukey’s HSD test. To evaluate the preventative effect of T-720 and biochar against each basidiomycete a Tukey’s analysis was also performed. The statistical analysis were performed using the statistical software SPSS^®^ (Version 22, SPSS Inc., Chicago, IL, USA).

## Results and discussion

### Antagonistic potential of *Trichoderma* against wood decay basidiomycetes

During initial screening of the *Trichoderma* species and strains a range of reactions were recorded as a result of antagonism. Contact between basidiomycetes and *Trichoderma* occurred in all cultures but the ability to overgrow and parasitize the mycelia of the basidiomycetes was dependent on the antagonistic potential of each *Trichoderma* and the resistance of the challenged fungi to antagonism (Table 3). The antagonistic potential of *Trichoderma* was most prevalent for T-720 showing very fast overgrowth for the studied basidiomycetes (Table 3). *S*. *himantioides* and *R*. *placenta* showed a moderate resistance to the other *Trichoderma* spp. *T*. *harzianum* (T-721), *Trichoderma atroviride* (T-722) and *T*. *koningiopsis* (T-723) revealed the weakest effect against the five basidiomycetes.

**Table 3:** Classification of the degree of mycoparasitism of *Trichoderma* species and strains against basidiomycetes.

Basidiomycetes	*Trichoderma*	*Trichoderma*	*Trichoderma*	*Trichoderma*	*Trichoderma*
	*atroviride*	*harzianum*	*harzianum*	*atroviride*	*koningiopsis*
	T-685	T-720	T-721	T-722	T-723
***Antrodia serialis***	+++^a^ (100)^b^	+++ (96)	+++ (84)	+++ (80)	+++ (100)
***Fibroporia vaillantii***	+++ (80)	+++ (96)	++ (60)	++ (64)	++ (76)
***Gloeophyllum sepiarium***	+++ (100)	+++ (100)	++ (80)	++ (60)	+++ (80)
***Rhodonia placenta***	+ (20)	+++ (100)	+ (40)	+ (60)	+ (20)
***Serpula himantioides***	++ (64)	+++ (100)	++ (56)	++ (60)	++ (64)

^a^; + = slow overgrowth, ++ = fast overgrowth, +++ = very fast overgrowth.

^b^; ability of *Trichoderma* to eradicate wood decay basidiomycetes (lethal effect in %) after 4 weeks in dual culture.

**Fig 1:**
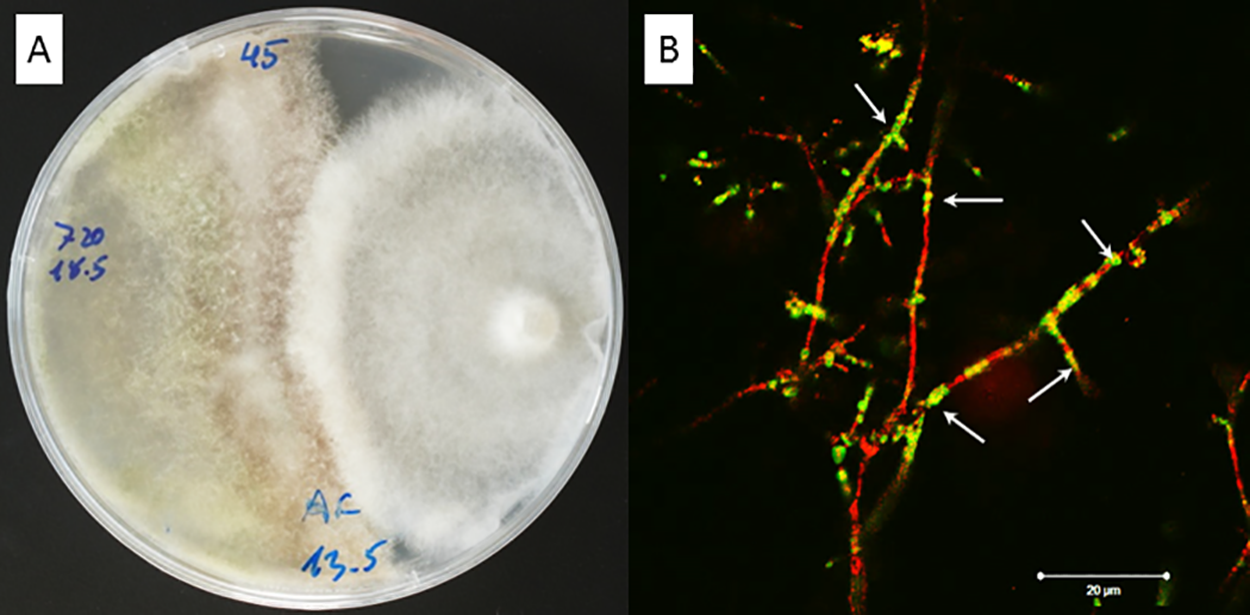
Dual culture test showing the antagonistic activity of T-720G against *R*. *placenta*. A: Rapid overgrowth of *R*. *placenta* by T-720G after 48 h. B: Mycoparasitism of T-720G (green) against *R*. *placenta* (red) after 48 h contact in dual culture.

The genetically transformed T-720G showed *in vitro* antagonistic activity similar to the parental strain (Fig 1A), and thus was used for further characterization of its biocontrol activity and colonization. After 48 h of contact between T-720G and *R*. *placenta*, overgrowth and development of typical parasitic colonization by *Trichoderma* on the target basidiomycete was observed. Confocal microscopy of these samples showed actively growing hypha of T-720G (Fig 1B, green) that became attached to the basidiomycete, surrounding it and generating structures similar to the appressoria-like structures (arrows in Fig 1B) as previously described by Harman et al. [36] and Schubert et al. [37, 38]. The lethality to the target fungus was confirmed by its staining with the cell death marker propidium iodide (Fig 1B, red). Additionally, the lethal effect demonstrated by the applied *Trichoderma* fungi in dual culture was highest for T-720 that recorded 100% deadlock within four weeks against *Gloeophyllum sepiarium*, *R*. *placenta* and *S*. *himantioides* and 96% deadlock against *A*. *serialis* and *Fibroporia vaillantii* (Table 3). *T*. *harzianum* (T-721) showed the weakest antagonistic potential against most of the basidiomycetes.

### *Trichoderma harzianum* (T-720) and oxalic acid production wood decay basidiomycetes

The data on the oxalic acid production by the wood decay basidiomycetes are shown in Table 4. Production of oxalic acid by the basidiomycetes *A*. *serialis*, *F*. *vaillantii*, *G*. *sepiarium* and *S*. *himantioides* was in good agreement with previous studies [13, 39]. Schmidt [40] showed most oxalic acid production by *Serpula lacrymans* followed by *Antrodia vaillantii* and *A*. *sinuosa*. Generally brown-rot fungi acidify their growth substrate more than white-rot species because the latter degrade the produced oxalic acid by oxalate decarboxylase to formate and CO_2_ [40]. *G*. *sepiarium* produced the highest amounts of oxalic acid for the control treatments (20.13 μg mL^-1^). However the production of oxalic acid by *G*. *sepiarium* did not show significant differences compared to *F*. *vaillantii* and *S*. *himantioides*. Results obtained for *R*. *placenta* (0.64 μg mL^-1^) showed a very low production of oxalic acid as previously demonstrated by Civardi et al. [13] and Ritschkoff et al. [39]. Studies by Ritschkoff et al. [39] on the oxalic acid production by *R*. *placenta* showed the most pronounced production (1 g L^-1^) after three weeks of cultivation. One week later, the oxalic acid content in the same cultures was significantly reduced (0.25 g L^-1^). Thus it seems that degradation of oxalic acid by *R*. *placenta* is also involved in the pathway of other oxidative reactions as hypothesized by Ritschkoff et al. [39]. Furthermore, the same tendency of decrease of oxalic acid measurements with time was observed for *Gloeophyllum trabeum* and *Coniophora puteana* by Hastrup et al. [41].

**Table 4:** Effect of T-720 on the oxalic acid (OA) production of wood decay basidiomycetes.

Basidiomycetes	Controls (μg OA mL^-1^)	Dual culture (μg OA mL^-1^)
***Antrodia serialis***	11.41(a) ± 2.14	7.61(a) ± 1.34
***Fibroporia vaillantii***	18.70(b) ± 1.22	9.40(ab)* ± 0.62
***Gloeophyllum sepiarium***	20.13(b) ± 0.32	11.54(b)* ± 0.37
***Rhodonia placenta***	0.64(c) ± 0.42	1.16(c) ± 0.46
***Serpula himantioides***	19.82(b) ± 0.65	8.53(a)* ± 1.04

*Significant reduction of oxalic acid production by T-720 treatment (t-test: *p*<0.05).

Different letters denote significant differences between the fungi after the Tukey’s HSD test (column-wise). Data represented as mean ± SD of three replicates.

The analysis of the supernatant in dual cultures showed that oxalic acid production was significantly reduced for *F*. *vaillantii*, *G*. *sepiarium* and *S*. *himantioides* compared to controls (*p*<0.05). After the T-720 treatment, *S*. *himantioides* showed the highest reduction of oxalic acid production (8.53 μg mL^-1^) compared to controls (19.82 μg mL^-1^). *G*. *sepiarium* produced the highest quantities of oxalic acid after the T-720 treatment (11.54 μg mL^-1^) but nevertheless the results were not statistically significant from the ones recorded for *F*. *vaillantii* (9.40 μg mL^-1^). Although *A*. *serialis* did not show significant reduction of oxalic acid production after the T-720 treatment (7.61 μg mL^-1^) compared to controls (11.41 μg mL^-1^), the production of oxalic acid in dual cultures was inferior than the production by *F*. *vaillantii* (9.40 μg mL^-1^), *G*. *sepiarium* (11.54 μg mL^-1^) and *S*. *himatioides* (8.53 μg mL^-1^). The effect of different biocontrol agents and organic biocides to reduce the production of oxalic acid has been previously demonstrated by some authors [42, 43]. For instance, Paramasivan et al. [43] demonstrated that the application of *T*. *viride* is a useful approach in controlling *Sclerotium rolfsii* in the soil by reducing more than three times the oxalic acid production (0.79 mg mL^-1^).

### Biochar and Cu-leaching from Cu-treated wood specimens

Our data showed that biochar binds Cu released during the leaching process of wood preservatives (Table 5). The amount of Cu released from treated wood was significantly higher in the case of Cr-free treated wood specimens (139.51 mg L^-1^ Cu-HDO and 52.51 mg L^-1^ ACQ) than in wood treated with Cr-containing formulations (17.57 mg L^-1^ CC and 28.71 mg L^-1^ CCB). Cu-adsorption by biochar was higher for Cr-free wood preservatives (95.0% Cu-HDO and 84.1% ACQ). However, significant differences between all wood preservative treatments were found after the Tukey’s test. The chemical composition of wood preservatives may also play an important role on the adsorption/desorption process of Cu due to the specific physical properties of biochar as previously demonstrated by Beesley et al. [23]. Chen et al. [44] demonstrated that the adsorption of Cu by biochar strongly correlates with pH, i.e. the highest adsorption of Cu occurred between pH = 4–8. Wood preservatives change the pH of a solution during the event of leaching. The Cu-retention by biochar from impregnated wood specimens in this study was in good agreement with other biochar products that are based on wood as demonstrated by Chen et al. [44] and Han et al. [45] (25.4–1.59 mg g^-1^). The Cu-retention capacity was strongly correlated with the source of the raw material used for producing biochar. For instance, studies by Tong et al. [46] showed a Cu-adsorption of 89.0 mg g^-1^ by Peanut straw and Pellera et al. [47], 0.27 mg g^-1^ by rice husks.

**Table 5:** Effect of biochar on Cu binding from Cu-treated wood specimens.

Treatment	Initial Cu in wood	Cu leached in water	Cu binding by biochar	Cu binding
	(mg g^-1^)	(mg L^-1^)	(mg g^-1^)	(%)
**Water control**	-	0.01(a) ± 0.00	0.02(a) ± 0.00	-
**Wood control**	0.02(a) ± 0.01	0.01(a) ± 0.00	0.02(a) ± 0.01	0
**CC**	5.04(b) ± 0.14	17.57(b) ± 0.01	3.67(b)* ± 0.16	72.8
**CCB**	5.90(c) ± 0.03	28.71(c) ± 0.01	2.71(c)* ± 0.25	45.9
**Cu-HDO**	9.07(d) ± 0.07	139.51(d) ± 0.07	8.62(d)* ± 0.11	95.0
**ACQ**	6.53(e) ± 0.01	52.51(e) ± 0.01	5.49(e)* ± 0.15	84.1

*Significant Cu binding by biochar (t-test: *p*<0.05).

Different letters denote significant differences between wood preservatives after the Tukey’s HSD test (column-wise). Data represented as mean ± SD of three replicates.

The change of the pH on the supernatant after 10 days in water solution is shown in Table 6. The pH of the water controls appeared to solubilise the wood preservative formulations when compared to the Cu-leached in solution (Table 5). The release of wood preservative compounds had a variety of influence on the pH values in solution. Wood specimens impregnated with CCB and ACQ preservatives did not alter the pH values significantly compared to controls. In contrast, CC and Cu-HDO demonstrated a significant increase in pH after 10 days in water. The addition of biochar significantly increased the pH values in all water solutions (Table 6). Higher differences were found on wood specimens impregnated with Cr-free preservatives (pH = 8.38 for Cu-HDO and pH = 8.08 for ACQ) compared to the treatments without biochar (pH = 6.88 and 5.64, respectively) and compared to the other wood preservatives after the Tukey’s test.

**Table 6:** Influence of biochar on the pH of water solution containing Cu-impregnated wood specimens.

Treatment	H_2_O	H_2_O + biochar
**Water control**	5.55(a) ± 0.04	6.81(a)* ± 0.01
**Wood control**	5.30(b) ± 0.11	7.06(b)* ± 0.06
**CC**	6.78(c) ± 0.04	7.30(b)* ± 0.01
**CCB**	5.72(a) ± 0.01	7.74(c)* ± 0.08
**Cu-HDO**	6.88(c) ± 0.04	8.38(d)* ± 0.16
**ACQ**	5.64(a) ± 0.13	8.08(e)* ± 0.08

*Significant increase of pH by biochar treatment (t-test: *p*<0.05).

Different letters denote significant differences between the fungi after the Tukey’s HSD test (column-wise). Data represented as mean ± SD of three replicates.

There are many factors that influence leaching of preservatives from wood into the soil such as exposure time, temperature, moisture content, inorganic ions or pH values [3]. The combination of all elements plays an important role on the amount of leachate in the field. For instance, Bergholm [48] demonstrated the correlation between the mobility of CCA components and the pH in the soil. Studies by Murphy and Dickinson [49] on the effect of acid rain on leaching of CCA-C, demonstrated that 40% of the Cu was lost at pH = 3, however, there was no significant loss of Cu at pH > 5.6. The wood preservatives used in this study are primarily in the form of Cu(OH)_2_ and CuCO_3_ that become more stable at pH around 7 [50–52].

### *Trichoderma harzianum* (T-720)-enriched biochar and wood mass loss reduction by wood decay basidiomycetes

**Fig 2:**
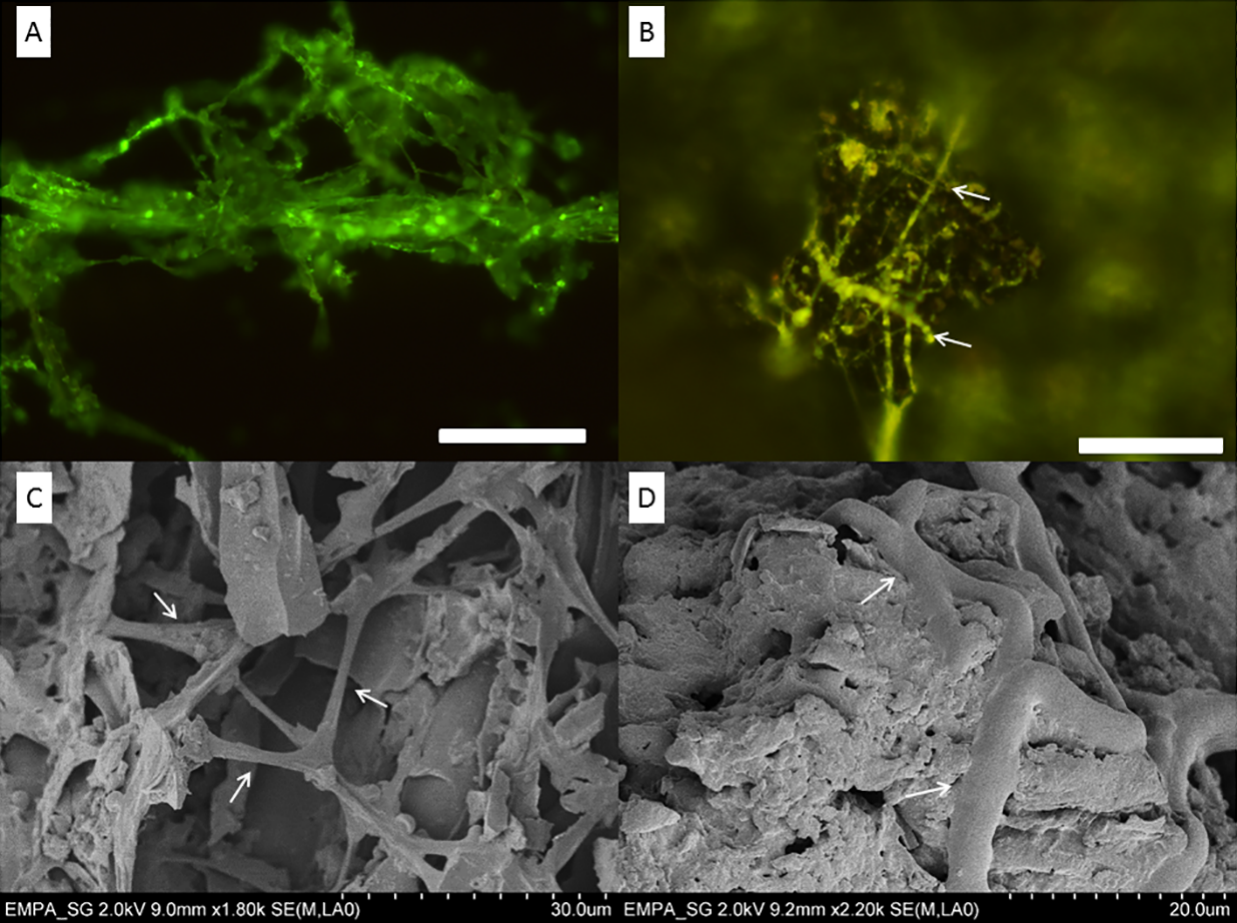
Colonization of biochar substrate by T-720 and T-720G after 48h. A: Transformed *T*. *harzianum* (T-720G). B: Colonization of biochar by T-720G. C and D: matrix created by hypha of T-720 in biochar. The scale bar in A and B represents 100 μm; in C and D represents 30 μm and 20 μm, respectively. Arrows: hyphae of T-720.

Microscopic interactions between T-720-biochar and T-720G -biochar revealed a rapid colonisation of the substrate by both strains. After 48 h incubation with T-720G (Fig 2A), almost all the biochar was colonised as observed under the fluorescence microscope (Fig 2B). Observations with SEM confirmed the behaviour of T-720 to develop a compact matrix between the biochar particles (Fig 2C and 2D). The addition of T-720 to the biochar substrate creates the opportunity to use biochar as a carrier substance for an integrated control strategy of wood decay basidiomycetes in soils.

The influence of biochar and T-720 treatments to protect wood is shown in the Table 7. The mass loss recorded by decay basidiomycetes was in the range of our previous studies [22]. The effect of T-720-enriched biochar on preventing mass loss was positively influenced for all basidiomycetes. However, the T-720-enriched biochar revealed a significant reduction of mass loss for *A*. *serialis* (0.51%), *G*. *sepiarium* (0.43%), *R*. *placenta* (0.37%) and *S*. *himantioides* (0.85%). Moreover, mass losses recorded in wood specimens placed into T-720-enriched biochar was below 3% of the initial dry mass for the basidiomycetes that is the adequate threshold recommended in the ENV 807 [30]. Although the treatment with biochar reduced wood decay by basidiomycetes, this treatment only showed a significant effect (*p*<0.05) on the mass loss caused by *S*. *himantioides* (2.28%) compared to controls (19.44%).

**Table 7:** Effect of T-720-enriched biochar on reducing wood mass loss caused by wood decay basidiomycetes.

Basidiomycetes	Control (%)	Biochar (%)	Biochar + T-720 (%)
***Antrodia serialis***	14.96(cdA) ± 2.10	12.57(dA) ± 0.82	0.51(aB) ± 0.13
***Fibroporia vaillantii***	2.78(aA) ± 2.66	1.15(aA) ± 0.29	0.62(aB) ± 0.19
***Gloeophyllum sepiarium***	6.90(abA) ± 0.80	6.04(abA) ± 0.38	0.43(aB) ± 0.15
***Rhodonia placenta***	11.86(bcA) ± 1.61	9.31(bcA) ± 3.10	0.37(aB) ± 0.09
***Serpula himantioides***	19.44(dA) ± 4.21	2.28(cdB) ± 1.29	0.85(aB) ± 0.46

Different lowercase letters denote significant differences between the fungi after the Tukey’s HSD test (column-wise). Different uppercase letters denote significant differences between the control, biochar and T-720-enriched biochar treatments after the Tukey’s HSD test (row-wise). Data represented as mean ± SD of three replicates.

The possibility to control wood decay by basidiomycetes in the laboratory has already been demonstrated by Ribera et al. [22]. In this previous study, T-720 demonstrated high antagonistic potential in combination with low concentrations of wood preservative formulations against basidiomycetes. The internal decay in wood poles is usually developed in the ground line and the application of T-720-enriched biochar in highly infected soils would reduce the damage by basidiomycetes. However, variation within species and regular application strategies should be considered to design further long-term studies in order to maintain the activity of *Trichoderma* in the field.

## Conclusions

We demonstrated the positive effect to use *Trichoderma harzianum* (T-720)-enriched biochar as integrated wood protection method against wood decay basidiomycetes in the laboratory. T-720 was confirmed as antagonistic strain demonstrating also significant reduction of oxalic acid by five brown-rot fungi. Reduction of the oxalic acid production around Cu-impregnated wood products could possibly enhance the efficacy of wood preservatives avoiding Cu-removal from the wood. It was also validated that in the absence of Cr in wood preservative formulations Cu leaching occurs rapidly and biochar can bind the released Cu from impregnated wood. The application of T-720-enriched biochar in combination with new generation of wood preservatives (Cr-free) may provide an additional value as a method of integrated wood protection in highly infested soils. Long-term field studies in collaboration with telecommunication companies from Switzerland and Germany are currently in progress to develop a suitable application strategy and confirm these results under natural conditions. Successful results in the field will help to develop a sustainable wood protection strategy to counteract damage by wood decay basidiomycetes in soils, prevent the unnecessary release of contaminants in the environment and ultimately extend the service life of wood products in ground contact.

## References

[1] British Standards Institution (BSI). EN 460 Durability of wood and wood-based products—Natural durability of solid wood—Guide to the durability requirements for wood to be used in hazard classes. London, United Kingdom; 1994.

[2] Freeman M, McIntyre C. A comprehensive review of copper-based wood preservatives with a focus on new micronized or dispersed copper systems. Forest Prod J. 2008; 58(11): 6–27.

[3] Lebow S. Leaching of wood preservative components and their mobility in the environment: summary of pertinent literature. Forest Products Laboratory General Technical Report. FPL-GTR-93. 1996; 36 pp.

[4] Taylor JL, Cooper PA. Effect of climatic variables on chromated copper arsenate (CCA) leaching during aboveground exposure. Holzforschung. 2005; 59: 467–472. 10.1515/HF.2005.077

[5] Bahmani M, Fromm J, Schmidt O, Melcher E. Residual metal content and metal distribution in chromium/copper-treated wood after field and laboratory leaching exposure. Eur J Wood Prod. 2015; 73: 377–384. 10.1007/s00107-015-0901-5

[6] Olaniran AO, Balgobind A, Pillay B. Bioavailability of Heavy Metals in Soil: Impact on Microbial Biodegradation of Organic Compounds and Possible Improvement Strategies. Int J Mol Sci. 2013; 14(5): 10197–10228. 10.3390/ijms140510197 23676353 PMC3676836

[7] Schmidt O, Moreth U. Biological characterization of *Poria* indoor brown-rot fungi. Holzforschung. 1996; 50: 105–110.

[8] Young GY. Copper tolerance of some wood rotting fungi. Report no. 2223 U.S. Department of Agriculture, Forest Service, Forest Products Laboratory, Madison, WI; 1961.

[9] Sutter HP, Jones EBG, Walchli O. The mechanism of copper tolerance in Poria placenta (Fr.) Cke. and Poria vaillantii (Pers.). Fr Mater Org. 1983; 18(4): 241–262.

[10] Murphy RJ, Levy JF. Production of copper oxalate by some copper tolerant fungi. T Brit Mycol Soc. 1983; 81: 165–168.

[11] Cervantes C, Gutierrez-Corona F. Copper resistance mechanisms in bacteria and fungi. FEMS Microbiol Rev. 1994; 14: 121–138. 8049096 10.1111/j.1574-6976.1994.tb00083.x

[12] Civardi C, Schwarze F, Wick P. Micronized copper wood preservatives: An efficiency and potential health risk assessment for copper-based nanoparticles. Environ Pollut. 2015a; 200: 126–132. 10.1016/j.envpol.2015.02.018 25705855

[13] Civardi C, Schubert M, Fey A, Wick P, Schwarze F. Micronized copper wood preservatives: efficacy of ion, nano and bulk copper against the brown rot fungus *Rhodonia placenta*. 2015b; 10, 11 PLoS One 10:e0142578 10.1371/journal.pone.0142578 26554706 PMC4640524

[14] Bollmus S, Rangno N, Militz H, Gellerich A. Analyses of premature failure of utility poles IRG/WP12-40584. International Research Group on Wood Protection; 2012.

[15] Ribera J, Schubert M, Fink S, Cartabia M, Schwarze F.W.M.R. Premature failure of utility poles in Switzerland and Germany related to wood decay basidiomycetes. Holzforschung. 2016; 71(3): 241–247. 10.1515/hf-2016-0134

[16] The European Parliament and the Council of the European Union. DIRECTIVE 98/8/EC OF THE EUROPEAN PARLIAMENT AND OF THE COUNCIL of 16 February 1998 concerning the placing of biocidal products on the market. Official Journal of the European Communities. 1998; L 123/1. http://data.europa.eu/eli/dir/1998/8/oj

[17] The European Parliament and the Council of the European Union. REGULATION (EC) No 1907/2006 OF THE EUROPEAN PARLIAMENT AND OF THE COUNCIL of 18 December 2006 concerning the Registration, Evaluation, Authorisation and Restriction of Chemicals (REACH), establishing a European Chemicals Agency, amending Directive 1999/45/EC and repealing Council Regulation (EEC) No 793/93 and Commission Regulation (EC) No 1488/94 as well as Council Directive 76/769/EEC and Commission Directives 91/155/EEC, 93/67/EEC, 93/105/EC and 2000/21/EC. Official Journal of the European Communities. 2006; 2006R1907. http://data.europa.eu/eli/reg/2006/1907/2014-04-10

[18] The European Parliament and the Council of the European Union. REGULATION (EU) No 528/2012 OF THE EUROPEAN PARLIAMENT AND OF THE COUNCIL of 22 May 2012 concerning the making available on the market and use of biocidal products. Official Journal of the European Communities. 2012; L 167/1. http://data.europa.eu/eli/reg/2012/528/oj

[19] Bruce A, King B, Highley TL. Decay resistance of wood removed from poles biologically treated with *Trichoderma*, Holzforschung. 1991; 45: 307–311.

[20] Highley TL, Ricard JL. Antagonism of *Trichoderma* spp. and *Gliocladium virens* against wood decay fungi. Mater Org. 1988; 23: 157–169.

[21] Brown HL, Bruce A, Staines HJ. Assessment of the biocontrol potential of a Trichoderma viride isolate. Part II: Protection against soft rot and basidiomycete decay. Int Biodeterior Biodegrad. 1999; 44: 225–231.

[22] Ribera J, Fink S, Bas MC, Schwarze FWMR. Integrated control of wood destroying basidiomycetes combining Cu-based wood preservatives and *Trichoderma spp*. 2017; PLoS ONE 12(4): e0174335 10.1371/journal.pone.0174335 28379978 PMC5381793

[23] Beesley L, Moreno-Jiménez E, Gomez-Eyles JL, Harris E, Robinson B, Sizmur T. A review of biochars’ potential role in the remediation, revegetation and restoration of contaminated soils. Environ Pollut. 2011; 159(12): 3269–3282. 10.1016/j.envpol.2011.07.023 21855187

[24] Lehmann J, Rillig MC, Thies J, Masiello CA, Hockaday WC, Crowley D. Biochar effects on soil biota–A review. Soil Biol Biochem. 2011; 43(9): 1812–1836. 10.1016/j.soilbio.2011.04.022

[25] Ameloot N, Graber ER, Verheijen FGA, De Neve S. Interactions between biochar stability and soil organisms: review and research needs. Eur J Soil Sci. 2013; 64: 379–390. 10.1111/ejss.12064

[26] Naár Z and Kecskes M. Factors influencing the competitive saprophytic ability of *Trichoderma* species. Microbiol Res. 1998; 53: 119–129. 10.1016/S0944-5013(98)80029-3

[27] Sieber TN. *Pynrenochaeta ligni-putridi* spp. nov., a new coelomycete associated with butt rot of *Picea abies* in Switzerland. Mycol Res. 1995; 99: 274–276.

[28] Schwarze FWMR, Jauss F, Spencer C, Hallam C, Schubert M. Evaluation of an antagonistic Trichoderma strain for reducing the rate of wood decomposition by the white rot fungus *Phellinus noxius*. Biol Control. 2012; 61: 160–168. 10.1016/j.biocontrol.2012.01.016

[29] European Committee for Standardization (CEN). EN 252 Field test method for determining the relative protective effectiveness of a wood preservative in ground contact. Brussels, Belgium; 1989.

[30] European Committee for Standardization (CEN). ENV 807 Wood preservatives Determination of the effectiveness against soft rotting micro-fungi and other soil inhabiting micro-organisms. Brussels, Belgium; 2001.

[31] Sesma A, Osbourn AE. The rice leaf blast pathogen undergoes developmental processes typical of root-infecting fungi. Nature. 2004; 431(7008): 582–586. 10.1038/nature02880 15457264

[32] Khang CH, Park SY, Rho HS, Lee YH, Kang S. Filamentous Fungi (*Magnaporthe grisea* and *Fusarium oxysporum*). Methods Mol Biol. 2006; 344: 403–420. 10.1385/1-59745-131-2:403 17033082

[33] Harries E, Gandía M, Carmona L, Marcos JF. The *Penicillium digitatum* protein *O*-mannosyltransferase Pmt2 is required for cell wall integrity, conidiogenesis, virulence and sensitivity to the antifungal peptide PAF26. Mol Plant Pathol. 2015; 16: 748–761. 10.1111/mpp.12232 25640475 PMC6638402

[34] Gandía M, Harries E, Marcos JF. The myosin motor domain-containing chitin synthase PdChsVII is required for development, cell wall integrity and virulence in the citrus postharvest pathogen *Penicillium digitatum*. Fungal Genet Biol. 2014; 67: 58–70. 10.1016/j.fgb.2014.04.002 24727399

[35] Chacón MR, Rodríguez-Galán O, Benítez T, Sousa S, Rey M, Llobell A, et al Microscopic and transcriptome analyses of early colonization of tomato roots by *Trichoderma harzianum*. Int Microbiol. 2007; 10(1): 19–27. 17407057

[36] Harman GE, Howell CR, Viterbo A, Chet I, Lorito M. *Trichoderma* species-opportunistic, avirulent plant symbionts. Nature Reviews. 2004; 2: 43–56. 10.1038/nrmicro797 15035008

[37] Schubert M, Fink S, Schwarze FWMR. Evaluation of *Trichoderma* spp. as a biocontrol agent against wood decay fungi in urban trees. Biol Control. 2008a; 45(1): 111–123. 10.1016/j.biocontrol.2008.01.001

[38] Schubert M, Fink S, Schwarze FWMR. *In vitro* screening of an antagonistic Trichoderma strain against wood decay fungi. Arboric J. 2008b; 31: 227–248. 10.1080/03071375.2008.9747541

[39] Ritschkoff AC, Rättö M, Buchert J, Viikari L. Effect of carbon source on the production of oxalic acid and hydrogen peroxide by brown-rot fungus *Poria placenta*. J Biotechnol. 1995; 40(3): 179–186. 10.1016/0168-1656(95)00044-Q.

[40] Schmidt O. Über die Porenhausschwämme. 20^th^ Holzschutztagung Rosenheim. 1995; 171–196.

[41] Hastrup ACS, Green F III, Lebow PK, Jensen B. Enzymatic oxalic acid regulation correlated with wood degradation in four brown-rot fungi. Int Biodeterior Biodegrad. 2012; 75: 109–114. http://doi.org/10.1016

[42] Dickman MB and Chet I. Biodegradation of oxalic acid: A potential new approach to biological control. Soil Biol Biochem. 1998; 30: 1195–1197. 10.1016/S0038-0717(98)00018-2

[43] Paramasivan M, Mohan S, Muthkrishnan N, Chandrasekaran A. Degradation of oxalic acid (OA) producing Sclerotium rolfsii (Sacc.) by organic biocides. Arch Phytopathol Plant Protect. 2013; 46(3): 357–363. 10.1080/03235408.2012.740983

[44] Chen X, Chen G, Chen L, Chen Y, Lehmann J, McBride MB, et al Adsorption of copper and zinc by biochars produced from pyrolysis of hardwood and corn straw in aqueous solution. Bioresour Technol. 2011; 102(19): 8877–8884. 10.1016/j.biortech.2011.06.078 21764299

[45] Han Y, Boateng AA, Qi PX, Lima IM, Chang J. Heavy metal and phenol adsorptive properties of biochars from pyrolyzed switchgrass and woody biomass in correlation with surface properties. J Environ Manage. 2013; 118: 196–204. 10.1016/j.jenvman.2013.01.001 23454371

[46] Tong X, Li J, Yuan J, Xu R. Adsorption of Cu(II) by biochars generated from three crop straws. Chem Eng J. 2011; 172: 828–834. 10.1016/j.cej.2011.06.069

[47] Pellera FM, Giannis A, Kalderis D, Anastasiadou K, Stegmann R, Wang JY, et al Adsorption of Cu(II) ions from aqueous solutions on biochars prepared from agricultural by-products. J Environ Manage. 2012; 96: 35–42. 10.1016/j.jenvman.2011.10.010 22208396

[48] Bergholm J. Studies on the mobility of arsenic copper and chromium in CCA-contaminated soil IRG/WP3571. International Research Group on Wood Protection; 1990.

[49] Murphy RJ and Dickinson DJ. The effect of acid rain on CCA treated timber IRG/WP3579. International Research Group on Wood Protection; 1990.

[50] McBride MB. Forms and distribution of copper in solid and solution phases of soil In: Loneragan JF, and others, eds. Copper in soils and plants. New York: Academic Press; 1981 pp. 25–45.

[51] Parker AJ. Copper in soils and plants In: Loneragan JF, and others, eds. Copper in soils and plants. New York: Academic Press; 1981 pp. 1–22.

[52] Baker DE. Copper In: Alloway BJ, ed. Heavy metals in soils. New York: John Wiley and Sons; 1990 pp. 151–176.

